# Complete Remission in Paralytic Late Tick-Borne Neurological Disease Comprising Mixed Involvement of *Borrelia, Babesia, Anaplasma*, and *Bartonella*: Use of Long-Term Treatments with Antibiotics and Antiparasitics in a Series of 10 Cases

**DOI:** 10.3390/antibiotics12061021

**Published:** 2023-06-07

**Authors:** Paul Trouillas, Michel Franck

**Affiliations:** 1 Centre Médical Péricaud, 69008 Lyon, France; 2 ADNucleis Biological Laboratory, 69290 Grézieu la Varenne, France; m.franck@adnucleis.com

**Keywords:** late tick-borne related neurological deficit, *Borrelia burgdorferi* sensu lato, *Babesia*, long-term treatment

## Abstract

This study aimed to demonstrate that severe neurological motor deficits in the context of late tick-borne disease with mixed microorganism involvement are eligible for long-term combined antibiotic/antiparasitic treatments. The inclusion criteria were: 1. neurological limb paralysis with a disability score >4 according to the EDSS Kurtzke disability scale; 2. serological tests pointing to an involvement of the main tick-borne microorganisms *Borrelia burgdorferi* s.l., *Babesia*, *Anaplasma*, and *Bartonella*; 3. a general disease for more than 6 months with fatigue, pain and subjective cognitive deficit. The patients were administered long-term treatments with repeated cycles (at least three) of 35-day IV ceftriaxone and repeated oral regimens of azithromycin–doxycycline and azithromycin–doxycycline–rifampicin. For *Babesia*, repeated courses of atovaquone–azithromycin were administered. Ten patients had intractable or severe motor deficits before treatment in the context of *Borrelia* (two cases) *Borrelia–Babesia* (four cases), *Borrelia–Babesia–Anaplasma* (two cases), *Borrelia–Babesia–Anaplasma–Bartonella* (one case) and *Babesia–Anaplasma* (one case). For several months, five had been in wheelchairs, and four had been walking with sticks. Seven patients out of 10 (70%) showed complete remission after a mean active treatment duration of 20.1 + 6.6 months, with a mean number of 4 ceftriaxone cycles. Three patients showed an initial remission but suffered secondary antibiotic/antiparasitic-resistant motor recurrences. Among the nine patients with *Borrelia* serologic positivity, treatments obtained complete remission in seven cases (77%). The findings of this ten-case series suggest the usefulness of long-term antibiotic/antiparasitic treatments in patients with severe late tick-borne neurological deficits with highly significant elements of tick-borne involvement.

## 1. Introduction

As opposed to early secondary neuroborreliosis, late neuroborreliosis is defined as a tertiary continuous neurological entity lasting more than 6 months [1,2,3,4,5,6,7,8]. Actually, some patients may show a several-year course of the disease. The entity corresponds to various central neuropathological features—particularly encephalomyelitis, encephalopathy, and meningovascular lesions and may or may not comprise peripheral involvement [[9], for review].

The antibiotic treatment of severe late Lyme neuroborreliosis is an unsettled issue. In the analysis of 16 antibiotic treatment studies of neuroborreliosis, only 15 patients had a late form, making scientific evaluation impossible [10] (see Appendix 3 in Attachment 3, Dersch et al., 2015). In the study of Hansen et al. [5], 8 patients with late neuroborreliosis had a particularly poor response to a single regimen of 10-day intravenous ceftriaxone 2 g/d: all 8 patients (100%) still had neurological symptoms after a 4–72-month follow-up (median 33). In a case series of 15 patients, only 3 (20%) were symptom-free after a sole 2–3-week ceftriaxone regimen of 2 g/d [11]. Thus, guidelines advising a single 2–3-week ceftriaxone regimen in late neuroborreliosis, on the basis of these data, appear insufficiently founded [12,13].

As a matter of fact, longer treatments have shown positive results in cognitive forms of late neuroborreliosis. In such an indication, Logigian et al. showed the cognitive benefit of a 30-day 2 g/d ceftriaxone regimen in 12/15 patients of a case study [14]. One patient of the series exhibited a cognitive relapse and recovered from it after a second 30-day ceftriaxone course. In a NIH-sponsored placebo-controlled trial of 37 patients with Lyme posttreatment encephalopathy [15], considered as belonging to “late Lyme disease” [16], Fallon et al. used a 70-day continuous treatment with ceftriaxone 2 g/d. The authors demonstrated a significant improvement in the cognitive index at week 12. Moreover, Oksi et al. successfully used a 149-day ceftriaxone administration on a patient having a deep brain lesion with biopsy-proven borreliosis [17]. Thus, the use of prolonged and repeated courses of IV ceftriaxone in late neuroborreliosis appears legitimate.

On the other hand, presumed “pure” late neuroborreliosis probably does not correspond to reality. Actually, tick-transmitted *Borrelia* diseases comprise infections mixed with microorganisms such as *Babesia*, *Anaplasma*, *Bartonella*, and others [18]. Severe neurological symptoms have been described in cases of proven *Borrelia–Bartonella* disease, requiring high doses and prolonged administration of various antibiotics [19]. Since *Babesia* is a frequent *Borrelia*-associated parasitosis [20,21,22], late neurological *Borrelia–Babesia* complex may be expected. *Anaplasma,* another classically associated microorganism [23], is also likely to be detected in this context. Such coinfections might require specific complementary treatments.

In this article, we describe a case series of 10 patients with severe neurological limb paralysis and mixed infections with *Borrelia*, *Babesia divergens*, *Anaplasma phagocytophilum*, and *Bartonella.* The treatment of these patients was exceptionally long and difficult. In 7 out of 10 cases, however, we were able to obtain a final complete motor recovery after a long-term administration of combined antibiotics and antiparasitics.

## 2. Patients and Methods

### 2.1. Clinical and Biological Inclusion Criteria

In this case series study, the required criteria were: 1. the existence of a late persistent neurological motor paralysis, isolated or multiple, with a score ≥4 evaluated by the 0–10 Kurtzke Disability Scale (EDSS) [24]; 2. The existence of a pre-neurological general disease comprising fatigue, pain, and subjective cognitive deficit, i.e., a “polymorphous post-tick-bite syndrome” (PPTS) for more than 6 months [25]; 3. Blood serological tests pointing to the involvement of at least one of the classically tick-related microorganisms: *Borrelia* sensu lato according to ELISA and/or immunoblot tests, *Babesia divergens*, *Bartonella henselae* or *quintana*, and *Anaplasma phagocytophilum*.

### 2.2. Follow-Up

Patients were examined every 3 months during the protocol, and every year after the end of protocol for 2 years.

### 2.3. Outcome Criterion

The end-of-study outcome criterion was the percentage of patients with a Kurtzke disability score of 0, i.e., a complete motor remission 2 years after end of protocol.

### 2.4. Neurological Analysis

All patients had a comprehensive neurological entry examination. The Kurtzke EDSS disability score [24] was determined at entry and every 6 months to evaluate the consequence of the paralytic state. The subjective permanent baseline pain burden, local or general, was evaluated from 0 to 10 at entry and every 6 months.

The following explorations were performed at entry: MRI with T2-weighted and FLAIR pulse sequence for encephalon and T2-weighted sequence for spinal cord; electromyogram; CSF: cell count, protein concentration, and search for an oligoclonal profile.

### 2.5. Infectious Involvement Criteria

All patients had a blood serology exploration at entry. For borreliosis, blood *Borrelia burgdorferi* sensu lato *(B. Burgdorferi* sensu stricto, *B. afzelii*, *B. garinii*, *B. spielmanii*), IgG-IgM ELISA tests were performed using the Diasorin CLIA technique for detecting *Bb*sl antibodies. The tests were scored positive when IgG concentration exceeded 15 UA/mL and IgM 22 UA/mL. IgG-IgM immunoblot using the All Diag Diasorin/Mikrogen technique was performed in order to detect antibodies to the following antigens of *Borrelia burgdorferi* sensu lato [26]: p100 (p83/100), VlsE, p58, p39, p31 (OspA), OspC Bss-Baf-Bga,-Bsp (p23), p18 Bss-Baf-Bba-Bg1-Bg2, BSp (p17 DbpA), p58 (OppA-2), and p41. Such bands corresponded to the ones described by Hauser et al. (1997) in European cases of borreliosis due to *Borrelia* strains sensu lato [27]. A supplementary search for p75 [28] and p22 [29] was performed in some cases. Quantitatively, the number of significant IgG and IgM bands was determined for each patient according to European interpretation of immunoblots [27]: p41 was included in the IgM specific band number and excluded from the IgG one. A two-tiered test was performed when possible. Patients with either ELISA positivity *or* the presence of immunoblot significant bands according to Hauser et al. (1997) [27] were considered as bearing significant *Borrelia* serological stigmata and included in the study.

Furthermore, particular qualitative attention was paid to the presence of antigens that have been found to correlate with neuroborreliosis: p18 [30,31]; p100, OspC [31,32,33]; and VlsE [32,33,34], a fact recently confirmed [35].

A serological indirect fluorescent antibody test (IFAT, Eurofins) was used to screen for the presence of antibodies against *Babesia divergens*, according to Chauvin et al. [36] and Lempereur et al. [37]. Serum samples were scored as positive at dilution 1/16, assigned the classical low antibody concentrations in humans, versus the high ones in animals. Initial *Babesia* positivity was an inclusion criterion. However, the repetition of IFAT tests in primarily *Babesia*-negative patients revealed secondarily positive *Babesia* tests, leading to targeted treatments against the parasite. *Babesia microti* testing was not available.

*Anaplasma phagocytophilum* serology was performed using Eurofins IgG and IgM Eurofins IFAT; tests were scored positive at dilution 1/332. *Bartonella* serology was tested for *Bartonella henselae* and *Bartonella quintana* with Eurofins IgG IFAT; positivity was also determined at dilution 1/332. Numerous other serological tests were performed for other microorganisms but were not considered as criteria for inclusion and specific treatments.

CSF *Borrelia* ELISA test and intrathecal *Borrelia* antibody synthesis were determined when lumbar puncture was possible.

### 2.6. Ruling Out of Other Diseases

Using an MRI, multiple sclerosis and transverse myelitis were ruled out by the absence of specific images. Autoimmune diseases such as neurolupus and Gougerot–Sjögren syndrome were eliminated on the basis of clinical symptoms and absence of specific antibodies. Guillain–Barré syndrome was excluded on the basis of CSF data and EMG.

### 2.7. Long-Term Treatment Regimens

General design: The basic design was to treat *continuously* until the disappearance of neurological symptoms, i.e., complete motor remission. In case of recurrence after remission, the treatment was resumed and continuously administered until possible further remission.

Anti-*Borrelia* treatments: All patients had a primary treatment consisting of a 35-day regimen of intravenous ceftriaxone 2 g/d with oral doxycycline 200 mg/d, followed by a 2–3-month oral regimen with doxycycline 200 mg/d-azithromycin 500 mg/d (DA) if possible. In case of incomplete primary results or recurrence, patients had at least 2 supplementary 35-day intravenous ceftriaxone cycles with oral doxycycline 200 mg, followed by doxycycline–azithromycin oral regimens if possible. Thus, all patients had at least 3 IV ceftriaxone cycles with oral doxycycline.

Beyond this point, incomplete results or recurrences with *Borrelia* stigmata had 2 types of regimens for several months until possible remission: either further repeated new ceftriaxone–oral doxycycline cycles and, if possible, doxycycline–azithromycin (DA) regimens, or a continuous treatment of doxycycline 200 mg/d-azithromycin 500 mg/d-rifampicin 300 mg/d (DAR).

Anti-Babesia treatments: a specific treatment using a 21-day regimen of atovaquone 1500 mg/d-azithromycin 500 mg/d (At-A) was administered in case of initial or secondary *Babesia* positivity; it was repeated in the presence of symptoms evocative of *Babesia*.

Anti-Anaplasma and anti-*Bartonella* treatments: patients with a positive serology for *Anaplasma phagocytophilum* and/or *Bartonella* serology were treated using a 3-month oral regimen of doxycycline 200 mg/d-azithromycin and 500 mg/d-rifampicin 300 mg/d, repeated in case of failure.

Probiotic-accompanying regimen—D3 vitamin supplementation: a daily regimen with 12-billion lactobacillus rhamnosus/d and 3000-units/d of D3 vitamin was administered during the active treatments.

Lines and inserted devices: For ceftriaxone courses, a daily venous puncture was requested. Several-day routine catheters were strictly forbidden. PICC lines and a portacath were proposed when necessary and performed by specialized medical professionals. Nurses used appropriate sterile technique while managing PICC lines for IV antibiotic administration.

## 3. Ethics Statement

The study obtained 22-942-IRB00003888 ethical approval from the CEE-IRB.

## 4. Results

### 4.1. Cohort Description and Patient Histories (Table 1)

Treatments, follow-up, and data analysis took place from July 2014 to March 2022. Ten patients were included in the series: eight women and two men. Three teenagers were included; the mean age was 29.8 ± 12 years. Six patients out of 10 had identified tick bites and 2/10 erythema migrans. The mean duration of pre-neurological general disease was 28.6 ± 25.9 months. At entry, cephalalgias were present in 9/10 patients. Symptoms compatible with *Babesia* infection—fever attacks, shivering, night sweats—were observed in 8/10 patients, matching with positive *Babesia divergens* serology in 6/8; stretch marks—possibly striae distensiae—were present in 6/10 patients and leucopenia in 2/10. With respect to previous antibiotic/antiparasitic treatments, all patients were naïve.

**Table 1:** Demographics and general symptoms at entry.

	1	2	3	4	5	6	7	8	9	10
**Age (years)**	13	35	25	16	14	68	49	40	34	27
**Gender**	F	F	F	F	F	F	M	M	F	F
**Identified tick bite**	+	+	0	+	+	0	+	0	0	+
**Erythema migrans**	+	0	0	0	0	0	0	0	0	+
**General disease duration at entry** **(months)**	6	20	10	65	21	36	84	12	12	20
**Fatigue**	+	+	+	+	+	+	+	+	+	+
**Subjective cognitive impairment**	+	+	+	+ #	+	+	+ #	+	+	+
**Cephalalgia**	+	+	+	+	+	+	+	+	0	+
**“*Babesia* symptoms”** **(night sweating, shivering, fever)**	+	+	+	+	0	+	+	+	0	+
**Stretch marks**	+	+	+	+	+	0	0	+	0	0
**Leucopenia**	+	0	0	0	0	+	0	0	0	0

# Cognitive impairment objectively demonstrated using tests.

### 4.2. Entry Neurological Status (Table 2)

All presentations consisted in persisting or aggravating limb paralysis, with a mean duration of 18.2 months (±19.3). The global disability of the patients was considerable, with a median Kurtzke disability score at 6 (range 4.5–8.5). Two patients had flaccid limb paralysis (case 1, tetraplegia; case 3, paraplegia), with depressed tendon reflexes and absence of the Babinski reflex. Five patients had a rather spastic pyramidal syndrome, with brisk reflexes, hypertonia but an inconsistent Babinski reflex. Five patients were in wheelchairs, while 4 patients were walking with sticks. The median baseline permanent pain burden was 8. A distal thermal hypoesthesia of the lower limbs was observed in 4 cases out of 10.

Socially, the situation was disastrous. All patients had interrupted school or work for several months or years, with a mean duration of school/work interruption of 18.8 ± 19.6 months.

**Table 2:** Neurological symptoms and social impact at entry.

	1	2	3	4	5	6	7	8	9	10
**Duration of neurological disease** **before treatment (months)**	6	13	6	48	9	16	60	6	6	12
**Kurtzke invalidity score**	8.5	6	8	7.5	7	7	6.5	6	6	4.5
**Flaccid tetraplegia**	+									
**Spastic triparesis**		+								
**Flaccid paraplegia**			+							
**Spastic paraplegia**					+					
**Spastic paraparesis**				+		+	+	+		
**Isolated limb palsy**									+	+
**Wheelchair before treatment**	+	-	+	+	+	+	-	-	-	-
**Walking with 1 or 2 stick(s)**	-	+	-	-	-	-	+	+	+	0
**Permanent pain score: 0–10**	8	8	9	9	8	6	9	8	5	7
**Interruption of work or school** **attendance**	+	+	+	+	+	+	+	+	+	+
**Duration of** **work (w)/school (s) attendance** **interruption** **before treatment (months)**	6 s	13 w	6 w	36 s	12 s	16 w	60 w	2 w	4 w	12 w

### 4.3. Entry Neurological Explorations (Table 3)

Brain and spine MRI was performed in 9 cases out of 10. Multiple T2-weighted image hyperintensities were observed in encephalon white matter in three cases and in the spinal cord in one case. The CSF was explored in 7 out 10 cases because of lumbar puncture refusal. Pleocytosis was observed in one case, hyperproteinorachia >0.45 g/L in one case, and oligoclonal IgG band pattern in two cases (n° 2 and 9). Lower limb EMG was negative for all tested patients.

**Table 3:** Summary of neurological explorations at entry.

	1	2	3	4	5	6	7	8	9	10
**MRI T2-weighted** **image hyperintensities:**	0	+	0	+	0	+	0	0	0	nd
**Encephalon Spinal cord**	0	0	0	+	0	0	0	0	0	nd
**Pleocytosis > 4 cells/cc**	0	0	0	0	nd	0	+	nd	0	nd
**Protein concentration > 0.45 g/L**	0	0	0	0	nd	0	+	nd	0	nd
**CSF Oligoclonal pattern**	0	+	0	0	nd	0	0	nd	+	nd
**Inferior limb EMG anomalies**	0	0	0	0	nd	0	0	0	0	0

### 4.4. Microorganism Involvement Status (Table 4)

The positive blood serologies for the cited microorganisms do not necessarily indicate active infection but provide useful information about their contacts with the subject and their probable involvement in the current disease.

**Blood**: Blood serological ELISA positivity for *Borrelia* was found in nine cases. Upon immunoblot, antigens from the four strains—*Borrelia Burgdorferi* s.s., *B. garinii, B. afzelii,* and *B*. *spielmanii*—were detected. The number of significant bands was increased in seven patients when adopting the criteria of Hauser et al.: p41 band included for IgM and excluded for IgG [27]. For instance, patient 5 had for IgG: OspCBaf, OspCBga; and for IgM: OspCBss, OspCBga, OspCBsp, p18Bga, p39, and p41; patient 10 had for IgM: p100, OspCBaf, OspCBga, OspCBsp, p18 Bg1, and p41. Two-tiered tests were positive in three cases.

Remarkably, p18—an antigen linked to *severe* neuroborreliosis [30]—was present in six cases. The antigens p100, OspC, and VlsE—linked to neuroborreliosis [31,32,33,34,35]—were respectively observed in three, three and two cases. Overall, 7 patients out of 10 had 1 or several neuroborelliosis-linked antigens among p18, p100, OspC, and VlsE.

*Babesia* positivity occurred in 8/10 cases (after repeated tests), *Anaplasma* in 4/10 cases (2 detected late), and *Bartonella* in 1 patient (n° 9); negativity for *Bartonella henselae* and *Bartonella quintana* did not rule out involvement of other *Bartonella* species.

**Table 4:** Summary of blood serological data.

	1	2	3	4	5	6	7	8	9	10
**ELISA *Borrelia* s. lato** **IgG**	0	+	+	+	0	+	0	0	0	0
**IgM**	0	0	0	+	0	+	0	0	+	0
**ELISA positivity**	0	+	+	+	0	+	0	0	+	0
**Immunoblot *Borrelia* s. lato IgG signif. band(s)**	0	±	+(5)	0	+(2)	+(2)	+(1)	+(1)	0	+(1)
**p41 excluded** **IgM signif. band(s)** **p41 included**	0	0	0	+(3)	+(6)	+(1)	0	0 *	0	+(6)
**Immunoblot positivity**	0	0	+	+	+	+	+	+	0	+
**Neuroborreliosis-correlated** **antigens (IgG/IgM)** **p18**	-	0	+	+	+	+	+	0	0	+
**p100**	-	0	+	0	0	0	0	+	0	+
**OspC**	-	0	0	+	+	0	0	0	0	+
**VlsE**	-	0	+	0	0	+	0	0	0	0
**Cumulated *Borrelia* stigmata (Bo)**	0	+	+	+	+	+	+	+	+	+
***Babesia divergens* (Bb)**	+	+	+	+	+	+	0	+	+	0
***Anaplasma* (A) IgG**	+	0	0	0	0	+	0	0	+	0
***phagocytophylum* IgM**	0	0	0	0	+	+	0	0	0	0
***Bartonella* (Bt)** ***henselae* IgG**	0	0	0	0	0	0	0	0	+	0
***quintana* IgG**	0	0	0	0	0	0	0	0	0	0
**Co-involvement status**		Bo	Bo	Bo	Bo	Bo	Bo	Bo	Bo	Bo
	Bb	Bb	Bb	Bb	Bb	Bb	-	Bb	Bb	-
	A				A	A			A	
									Bt	
**Intrathecal *Borrelia*** **antibody synthesis**	0	0	0	0	-	0	0	-	0	-

* IgM had been clearly positive 7 years ago. Therefore, the patients may be categorized in the following groups: *Borrelia* 2 cases: n° 7 and 10. *Borrelia-Babesia* 4 cases: n° 2, 3, 4, 8. *Borrelia-Babesia-Anaplasma*: 2 cases, n° 5 and 6. *Borrellia-Babesia-Anaplasma-Bartonella*: 1 case: n° 9. *Babesia–Anaplasma*, 1 case: n° 1. **CSF:** Intrathecal synthesis of *Borrelia* antibody was negative in all tested patients.

### 4.5. Realization of the Long-Term Treatments (Table 5)

The mean number of cycles with ceftriaxone–oral doxycycline was 4.2 ± 1.8. (range: 3–8), doxycycline–azithromycin 3.1 ± 0.9, doxycycline–azithromycin–rifampicin 1.8 ± 2.1, and anti-*Babesia* atovaquone–azithromycin 2.9 ± 1.3. The mean active treatment duration to first remission was 13.1 ± 10 months and to protocol end 20.1 ± 6.6.

**Table 5:** Summary of long-term treatment data.

	1	2	3	4	5	6	7	8	9	10
**Number of ceftriaxone–oral doxycycline cycles**	3	8	4	4	3	7	3	4	3	3
**Number of 2–3-month** **doxycycline–azithromycin c.**	1	4	4	3	3	4	4	3	3	2
**Number of 2–3-month** **doxy–azithro-rifampicin c.** **(possibly continuous)**	3	0	0	3	4	0 *	3	5	0 *	0
**Number of atovaquone–** **azithromycin cycles**	4	5	4	1	2	3	2 **	4	3	1
**Duration of treatment to** **first motor remission** **(months)**	8	4	3	23	12	10	26	31	4	10
**Cumulated duration** **of active treatment** **(months)**	18	19	19.5	23	26	22 ***	26	31	14.5	10

* Lately discovered *Anaplasma* infection could not be treated specifically. ** These anti-*Babesia* treatments were administered on the basis of symptoms suggestive of *Babesia* infection, although *Babesia* serology was negative. *** The treatment was interrupted because of administrative opposition.

### 4.6. Lines and Inserted Devices

PICC lines were used in three cases and a portacath in one case.

### 4.7. Treatment Outcomes (Table 6)

Final and durable complete motor remission (Kurtzke score 0), verified at 2 years, was observed in 7 out 10 cases (70%) after a mean treatment duration of 20.1 ± 6.6 months. In this successful subgroup of patients, the mean number of ceftriaxone cycles was 4 ± 1.8 and the mean duration of active treatment to complete motor remission was 20.6 ± 7.7 months. In three patients, (n° 2, 5 and 9), complete remission was obtained despite motor recurrences.

**Table 6:** Summary of treatment outcomes.

	1	2	3	4	5	6	7	8	9	10
**Complete motor remission** **at 2 years after treatment end**	0	+	0	+	+	0	+	+	+	+
**Kurtzke** **Invalidity score** **at 2 years after** **treatment end8**	8	0	8	0	0	8	0	0	0	0
**Kurtzke Ivalidity** **Score at entry** **(reminder)**	8.5	6	8	7.5	7	7	6.5	6	6	4.5
**Wheelchair after treatment**	+	0	+	0	0	+	0	0	0	0
**Number of motor recurrences** **during treatment course**	1	6	2	0	1	4	0	0	1	0
**Cumulated duration of c.** **active treatment in successful cases** **(months)**	-	19	-	23	26	-	26	31	14.5	10
**Time from treatment onset** **to final motor remission (months)**	-	41	-	23	36	-	26	31	17	10
**Return to work or school**	0	+	0	+	+	0	+ &	+	+	+
**Cumulated duration** **of work(w)/school (s)** **interruption** **in successful patients** **at final evaluation (months)**	-	54 w	-	54 s	14 s	-	87 w	33 w	10 w	16 w

**&:** This truck driver finally returned to truck driving.

A general observation was that each treatment cycle—particularly those of ceftriaxone—resulted in a quantum of partial improvement, so that repeated treatments were obviously necessary to achieve complete remission.

In the more specific subgroup with *Borrelia* serologic positivity, seven out of nine patients had final remission (77%).

The human and social consequences of remission were spectacular: a return to school or work in all seven cases. However, the life-destroying aggression experienced by these patients remained considerable, with an overall mean cumulative school or work interruption of 38.3 ± 28.2 months.

Three patients (n° 1, 3, and 6) had initial complete remission, enabling treatment to be stopped primarily. However, recurrences occurred (4 in patient n° 6), that appeared resistant to further treatment cycles, resulting in final disability.

### 4.8. Side Effects

Under the permanent protecting probiotic regimens, no major side effect—especially *Clostridium difficile* infection—was observed in the cohort. Benign candidiasis episodes were observed in three patients: oral in three cases and vaginal in one case; they were easily controlled with conventional general and local antifungal treatments. No side event took place in relation with PICC lines or the portacath. Repeated PICC line thrombosis forced the device to be changed 3 times in patient 2.

## 5. Discussion

### 5.1. High Rate of Complete Remission with Long-Lasting Treatments in a Cohort with Intractable Neurological Deficits and Significant Elements of Tick-Borne Involvement

The study resulted in a global 70% rate of complete remission with long-term treatments in a cohort defined by precise criteria: (i) severe neurological symptoms comprising limb paralysis; (ii) a general disease comprising fatigue, pain, and subjective cognitive deficit; (iii) blood serological positivity pointing to the involvement of classical tick-borne microorganisms: *Borrelia* sensu lato according to ELISA and/or immunoblot tests, *Babesia divergens, Bartonella henselae* or *quintana*, and *Anaplasma phagocytophilum*.

In other words, a decision to treat was considered as legitimate, and turned out to be successful facing severe neurological symptoms and clinico-biological data considered as significant elements of tick-borne involvement.

In more precise biological terms, complete regression was obtained in seven patients out of nine who had positive *Borrelia* ELISA (77.7%). Out of the seven patients with the documented neuroborreliosis-linked antigens p18, p100, OspC, and VlsE [30,31,32,33,34,35], i.e., patients with molecular indicators of highly probable neuroborreliosis, five were cured (71.4%).

Given the fact that the severe neurological deficits were clinical markers for a subjacent hypothesized tick-borne infectious disease, their high-rate disappearance under antibiotic treatment appears consistent with their infectious origin.

### 5.2. Long-Term Treatment Aspects and Results

The realization of ultra-long-term treatments in neuroborreliosis was originally performed by Oksi et al. in 1996 [17]; the authors successively administered to patient n° 2 a 2 g/d-ceftriaxone treatment for 21 days, 28 days, and 100 days, i.e., 149 days. Given the observation of coexisting bacterial and parasitic involvements, the current protocol proposed the combination of long-term ceftriaxone courses and adapted treatments against the other microorganisms.

Long-term antibiotic/antiparasitic protocols resulted in 7 complete neurological remissions out of 10 cases (70%) after a mean treatment duration of 20.1 + 6.6 months. In the successful subgroup of 7 patients, the mean number of ceftriaxone cycles was 4 ± 1.8 and the mean duration of active treatment to complete motor remission was 20.6 ± 7.7 months.

The global tolerance of the treatment was good thanks to the permanent accompanying probiotic regimen. Thanks to a meticulous management, the PICC lines and portacath did not generate complications. Thus, severe adverse effects were not observed. In this regard, the benefit/drawback ratio of the current long-term treatments appears clearly positive.

#### 5.2.1. Ceftriaxone Administration Duration and Remission

In the specific subgroup with *Borrelia* serological positivity, the complete remission rate was 7/9 (77%) with treatments comprising a mean number of 4 successive 35-day-ceftriaxone courses, while published single ceftriaxone 10–21-day short protocols resulted in 20% response or less [5,10,11]. Thus, a mean cumulative 140-day duration of ceftriaxone is likely to play a major role in complete remission.

The objective partial improvements in limb deficits observed after each cycle—especially ceftriaxone—explain why repeated cycles were necessary to clear the deficit completely in the successful cases.

Such successes of long-term treatments are in line with the Oksi et al.’s 1996 study [17], in which patient n° 2, with biopsy-proven brain lesions with *Borrelia,* was finally cured after the 149-day ceftriaxone protocol.

The idea that a neural eradication of *Borrelia* might be achieved with a single 2–3-week ceftriaxone regimen does not appear as scientifically tenable. Embers et al. showed that a single 30-day IV ceftriaxone cycle, even though followed by a 2-month doxycycline regimen, does not eradicate *Borrelia* from macaque brains [38]. Even a long-term (70-day) ceftriaxone regimen, as used by Fallon et al. in a controlled study about late neuroborrelian encephalopathy [15], showed a significant effect at week 12, but it was not sustained at week 24. Such a partial effect may be due to an insufficient cumulative ceftriaxone duration.

In this regard, future studies on late neuroborreliosis might reasonably take into account the “long-term ceftriaxone strategy” upon which the current study was actually built.

A synergistic effect of oral doxycycline relative to IV cephalosporin may also be discussed, since such a role has been demonstrated in vitro on *Borrelia* persister forms [39,40]. Yet, such a benefit might also be due to a therapeutic action on coinfection.

#### 5.2.2. Importance of Coadministration of Doxyxycline, Azithromycin, and Rifampicin for *Borrelia* and *Anaplasma*

The use of repeated cycles of 2–3-month doxycycline–azithromycin (mean number: 3.1) and of doxycycline–azithromycin–rifampicin (mean number: 1.8) in addition to ceftriaxone cycles may also have played a role in neurological remission, i.e., in the probable neural destruction of *Borrelia* and *Anaplasma*. Interestingly, in Oksi et al.’s index study, referring to cured patient n° 2, a 21-day rifampicin 600 mg/d regimen and a 21-day azithromycin 250 mg/d regimen were administered on top of IV-ceftriaxone in the first phase of the treatment [17].

#### 5.2.3. Importance of Coadministration of Repeated Anti-*Babesia* Atovaquone–Azithromycin Regimens

The taking into account *Babesia* involvement in *Borrelia* cases—that led to a mean number of 2.9 atovaquone–azithromycin treatments—may have participated in the high rate of complete remission in the 6 patients with *Babesia* out of 8 cases also with *Borrelia*.

Such results lead to a reconsideration of published studies that treated borreliosis without taking into account coinfection with *Babesia* [41,42]. Their negative results may be due not only to insufficient duration of anti-*Borrelia* treatments, but also to non-treatment of *Babesia* infection.

### 5.3. Treatment Decision and the Nosological Situation of the Cohort

The nosological situation of these patients might be that of “late neurological tick-borne disease”, a describing tool that encompasses highly probable neuroborreliosis in most cases and takes into account the mixing of microorganism involvement.

#### 5.3.1. Borrelian Involvement

In such a framework, borreliosis appears as the dominating background because nine patients had positive *Borrelia* s.l. ELISA tests. Rich immunoblot bands and two-tiered tests lead to a probable diagnosis of borreliosis in 7/9 cases.

Regarding neuroborreliosis, no *Borrelia* antibody synthesis was found in tested cases, while CSF biological abnormalities comprised slight hyperproteinorachia and pleocytosis in only two cases.

Yet, importantly, seven patients out of nine had neuroborreliosis-linked specific antigens p18, p100, OspC, and VlsE [30,31,32,33,34,35]. Six out of nine had p18, an antigen linked to severe neuroborreliosis [30]. This means that the combination of intractable neurological deficits and *Borrelia* ELISA/immunoblot positivity circumscribed a population of patients with a high prevalence of neuroborreliosis-linked antigens, i.e., molecular indicators of highly probable neurogenic *Borrelia* involvement.

At the diagnostic level, these data are rather in keeping with the recommendations of the American Academy of Neurology [43], that—even in their 2021 version [44]—do not include CSF data in the necessary criteria of neuroborreliosis diagnosis, while European recommendations do [45].

#### 5.3.2. Mixed *Borrelia*–*Babesia* Involvement

On the other hand, the combination of intractable neurologic deficits and *Borrelia* serological positive serologies circumscribes a population of patients with an 80% chance of having positive *Babesia* serology. Out of nine patients with *Borrelia* ELISA-positive tests, seven had positive *Babesia* serology. These data reveal the intermingling of *Borrelia* and *Babesia* involvements in these late neurological tick-borne cases. This nosological fact makes a specific accompanying anti-*Babesia* treatment rational.

#### 5.3.3. Non-*Borrelia* Involvement, with *Babesia*–*Anaplasma* Positivity

Patient 1 embodies a case of severe neurological deficit with heavy tick-borne context (13 simultaneous tick-bites), without apparent positive *Borrelia* data. Although this 13-year-old teenager had a complete primary neurological response to the protocol, she experienced relapses and a final failure.

#### 5.3.4. Low Rate of MRI Neural Hyperintensities

The inconstancy of MRI white-matter hyperintensities in neuroborreliosis was observed in 8 studies, ranging from 15% to 63% [2,46,47,48,49,50,51,52]. Thus, MRI images may not be taken as criteria for neuroborreliosis diagnosis and treatment. Our results are in keeping with the literature.

#### 5.3.5. Treatment Decision Basis

None of the 10 patients would have been accepted in a protocol using CSF criteria of late neuroborreliosis. Thus, seven neurological recoveries would have been missed. In this regard, the broadening of the clinico-biological criteria for late tick-borne neurological manifestations results in an increased capacity of therapeutic intervention facing late grave neurological symptoms. CSF and MRI data appear as noncompulsory criteria for treatment decision. Such a position had already been adopted twice by Logigian et al. (1990, 1999) [4,14], who had decided to treat Lyme encephalopathy patients, although they lacked CSF *Borrelia* antibodies, respectively, in 54% and 38.8% of cases. Patient 2 of Oksi et al. [17], who was treated and cured after 149 days of IV ceftriaxone, had no CSF *Borrelia* antibodies.

Overall, this series shows that long-term treatments have a 70% chance of success with a treatment decision based on a sufficient nosological basis, combining profound neurological deficits and significant clinico-serological elements of tick-borne involvement.

### 5.4. Responsibility of Coinfection in the Severity of the Neurological Status

The seven cases with mixed *Borrelia*–*Babesia* involvement raise the problem of the participation of *Babesia divergens* in palsies, while a neurological role for *Babesia* was hitherto only known in confusion and coma [53]. Such a deleterious pathogenetic role might be in keeping with the aggravation brought by *Babesia* described by Krause et al. [21].

The severity of paralysis in three patients with mixed *Borrelia–Babesia–Anaplasma* and one with *Babesia–Anaplasma* might be explained by the neurological tropism of *Anaplasma* [54,55]. In patient n° 9 with *Bartonella* (n° 9), the neurotropic pathogenicity of *Bartonella henselae* may also be discussed [56].

### 5.5. Factors of Failure

In patients 3 and 6, who had a poor final outcome, the simultaneous presence of p18 and VlsE was observed. Despite the fact that p18 was considered as a marker of particularly severe neuroborreliosis [30], the other patients with p18 without VlsE (four out of six) had a good final outcome. Thus, the molecular combination of p18 and VlsE might be a factor of resistance to antibiotic treatment.

In patient 1, who had no *Borrelia* elements and a remarkable primary response to treatment, no explanation can be provided for secondary resistance.

### 5.6. Indications for Future Studies

Given the mixed microorganism involvement observed in this series, it would be reasonable to have future therapeutic studies for late neurological tick-borne cases with a previous complete precise determination of the global infectious status and adapted antibiotic/antiparasitic regimens. Thus, we welcome future controlled studies that will rigorously distinguish several nosological subgroups of late tick-borne neurological disease: (i) “pure” late neuroborreliosis, without any co-infection, actually a possibly rare form; (ii) late neuroborreliosis with *Babesia,* a frequent form; (iii) late neuroborreliosis with *Babesia* and *Anaplasma;* (iiii) non-*Borrelia* late tick-borne disease with *Babesia* and *Anaplasma*, etc. Each of these subgroups should have a long-term treatment plan corresponding to the specific microorganism involvement.

### 5.7. Moral and Economic Considerations

Long-term protocols require great effort from patients and sustained monitoring by physicians. Their cost is also considerable and may multiply by 50 to 100 times the therapeutic financial investment when compared to habitual short treatments.

However, what price-tag can one put on the benefit of the return of an adult person to complete motor autonomy and to work? Moreover, on that of a teenager to normal life? These human advantages are enormous. The cured patients, after having experienced a long neurological nightmare, shared moving testimonies about the dignity of their new life. Patient 8 returned to his supermarket director’s job after a 33-month interruption and decided to have a baby with his partner. The baby duly arrived.

From a strictly economic point of view, the social cost of permanent disabled persons with late severe tick-borne neurological involvement may be compared with the cost of disabled patients with progressive multiple sclerosis, who have comparable high Kurtzke disability scores. Such a financial cost is considerable, and its annual burden may be over USD 70,000 [57]. Final complete remission, chiefly when enabling a return to work, represents an enormous economic benefit.

Such therapies are not only morally good actions, but also highly socially profitable enterprises.

## 6. Limitations

This study has some limitations. It is a case series, not a trial, and only suggests scientific conclusions. The number of patients was not high. The criteria for ruled out diseases—neurolupus, Gougerot–Sjögren syndrome, multiple sclerosis, Guillain–Barré syndrome—might be discussed. Concerning the biological tests, while the CLIA *Borrelia sensu lato* ELISA tests are largely diffused, specific IFAT tests for *Babesia*, *Anaplasma Phagocytophilum*, and *Bartonella* may be more specific and difficult to reproduce. Immunoblot interpretation might also be discussed, since the analysis criteria are different in Europe, in the U.S., and in China.

## 7. Conclusions

The findings of this ten-case series suggest that the use of long-term antibiotic/antiparasitic treatments is useful in patients with severe late neurological deficits coexisting with highly significant elements of tick-borne involvement.

## Data Availability

Complementary data may be accessed directly from investigators by email.
